# Effects of extracorporeal shock wave therapy in patients with knee osteoarthritis: A cohort study protocol

**DOI:** 10.1097/MD.0000000000021749

**Published:** 2020-08-28

**Authors:** Xianfei Xie, Jialing Zhu, Hao Zhang

**Affiliations:** a Department of Traumatology, Ruijin Hospital, Shanghai Jiao Tong University School of Medicine; b Department of General Family Medicine, Ouyang Community Health Service Center in Hongkou District of Shanghai, Shanghai, China.

**Keywords:** extracorporeal shockwave therapy, knee osteoarthritis, pain control, protocol, retrospective

## Abstract

**Background:**

Osteoarthritis is the most common form of arthritis, and is a major cause of disability and chronic pain in adults. However, there is very limited evidence in the scientific literature to support the effectiveness of extracorporeal shockwave therapy (ESWT) in human knee osteoarthritis. This retrospective study aimed to compare the efficacy of ESWT treatment with sham-ESWT on pain, walking speed, physical function, and adverse effects in knee osteoarthritis.

**Methods:**

This study will be performed and reported in accordance with the Strengthening the Reporting of Observational studies in Epidemiology checklist. We reviewed patients diagnosed with knee osteoarthritis at our academic center from 2016 to 2017. This retrospective cohort study was approved by the institutional review board in Ruijin Hospital. The primary outcome measure was pain on movement measured by a 100-cm visual analog scale. The secondary outcome measures included the Western Ontario and McMaster University Osteoarthritis Index, range of motion, and adverse effects. Statistical analysis was performed using Statistical Package for Social Sciences version 20.0 (IBM Corporation, Armonk, NY). A *P*-value of <.05 was defined as statistical significance.

**Results:**

The hypothesis was that ESWT would be an effective treatment for improving pain and physical function in knee osteoarthritis to control symptoms.

**Trial registration:**

This study protocol was registered in Research Registry (researchregistry5801).

## 1 Introduction

Osteoarthritis is the most common form of arthritis, and is a major cause of disability and chronic pain in adults.^[1,2]^ Even though osteoarthritis can involve single and/or multiple peripheral joints, including the knee, hip, and hand, the knee is the most common joint localization of symptomatic osteoarthritis.^[2]^ Knee osteoarthritis is the most common cause of disability and joint pain in adults,^[3,4]^ and is primarily characterized by chronic pain which is exacerbated by central sensitization,^[5,6]^ and reduced physical functioning. In addition, patients with osteoarthritis often suffer from comorbid depression^[7]^ and anxiety, and significantly worse quality of life.^[8,9]^

Conservative treatment has been regarded as the first-line therapy for knee osteoarthritis, including rest, nonsteroidal anti-infammatory drugs, physiotherapy, corticosteroid injection, or dry needling.^[10–12]^ However, the effectiveness of these treatments is still not well-established. Extracorporeal shockwave therapy (ESWT) appears to be a promising alternative and has been proven to be beneficial in several musculoskeletal diseases and especially enthesopathies, including plantar fasciitis, elbow epicondylitis, patella tendinitis, and achilles tendinitis.^[13,14]^ ESWT is a pulsed sound wave, characterized by short duration, high pressure amplitude, and relatively low tensile wave component.^[15]^ The mechanism of ESWT is not completely clear. However, it is speculated that ESWT may produce a reflexive analgesic effect by inducing excitability of the axon and destroying unmyelinated sensory fibers.^[16]^

Although there are many studies about ESWT on musculoskeletal disorders as well as on chondroprotective effect on animal have been published,^[17–19]^ there is very limited evidence in the scientific literature to support the effectiveness of ESWT in human knee osteoarthritis.^[20]^ Thus, more studies are still needed to explore the effect and safety of ESWT on the treatment of knee osteoarthritis. This retrospective study aimed to compare the efficacy of ESWT treatment with sham-ESWT on pain, walking speed, physical function, and adverse effects in knee osteoarthritis. The hypothesis was that ESWT would be an effective treatment for improving pain and physical function in knee osteoarthritis to control symptoms.

## 2 Materials and methods

This study will be performed and reported in accordance with the Strengthening the Reporting of Observational studies in Epidemiology checklist. We reviewed patients diagnosed with knee osteoarthritis at our academic center from 2016 to 2017. This retrospective cohort study was approved by the institutional review board in Ruijin Hospital (SHRJ00127) and was registered in the research registry (researchregistry5801).

### 2.1 Patients

Inclusion criteria: patients diagnosed with knee osteoarthritis according to the diagnostic criteria of American College of Rheumatology (ACR) who wanted to receive shock wave therapy. ACR criteria included that of knee pain, osteophytes, and 1 of the following: age >50 years, morning stiffness <30 minutes duration, or crepitus on active motion of the knee. The enrolled patients were over 45 years old, with unilateral knee joint symptoms, knee pain in the past 3 months, K-L classification of grade 2 or 3, and cartilage magnetic resonance imaging diagnosis of Recht grade II or III. Exclusion criteria: patients with bilateral knee joint symptoms; patients with a history of spinal stenosis; patients with a history of nervous system disease or secondary arthritis (inflammatory or metabolic); patients receiving surgery in the involved knee joint or intra-articular injection within the past 6 months; and patients with any contraindication of magnetic resonance imaging or radioscopy.

### 2.2 Interventions

Participants in the experimental group received ESWT. All ESWTs were given by a single experienced physical therapist. ESWT was conducted using an Electro Medical Systems instrument once a week for 4 consecutive weeks (4 sessions in total). The parameters of therapy included a total of 2000 pulses of 8 Hz frequency at 2.5 bar of pneumatic pressure. The first 1000 pulses were evenly distributed to pain points (the maximum number of pain points is 4). The remaining pulses were slid back and forth on the patellofemoral and tibiofemoral borders. No local anesthesia or other injections was used. Participants assigned to the placebo group were managed by the same physical therapist with the same ESWT protocol, but the air pressure was set at 0.2 bar.

### 2.3 Home exercise

As part of the treatment program, all participants, regardless of the group, were educated on a simple home exercise program for the first visit. The program was comprised of a single knee extensor muscle strengthening. The patient sat in a chair, straightened his/her knee as far as possible, kept it for 10 seconds, repeated 10 times, and did 3 groups per day. Therapist-applied manual forces were not permitted in the exercise program. The home exercise was supervised by a physiotherapist once every 3 days over the phone.

### 2.4 Outcome evaluation

The patient demographics were recorded retrospectively from their electronic patient notes. The primary outcome measure was pain on movement measured by a 100-cm visual analog scale (VAS). Pain was evaluated on a VAS with 0 = no pain, and 100 = worst imaginable pain. The secondary outcome measures included the Western Ontario and McMaster University Osteoarthritis Index (WOMAC), range of motion, and adverse effects. The WOMAC assesses symptoms of osteoarthritis and is a validated disease-specific self-reporting questionnaire referring to the 48 hours before assessment. The index consists of 5 questions for severity of knee pain, 2 for stiffness, and 17 for limitations in physical function. The WOMAC score ranges from 0 (best) to 96 (worst), with high score representing worse symptom severity (Table 1).

**Table 1:**
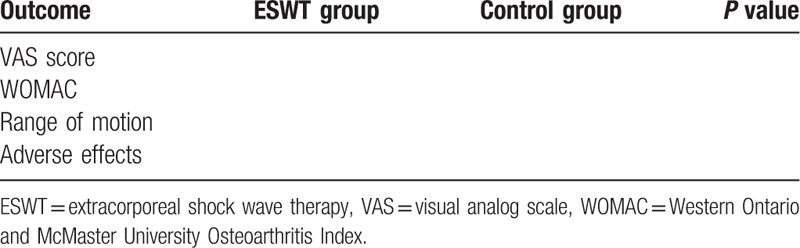
Postoperative outcomes.

### 2.5 Statistical analysis

Statistical analysis was performed using Statistical Package for Social Sciences version 20.0 (IBM Corporation, Armonk, NY). Parametric and non-parametric tests were used as appropriate to assess continuous variables for significant differences between groups. A Student *t* test was used to compare linear variables between groups. Dichotomous variables were assessed using a chi square test. Multivariate linear and regression analyses were used to identify independent predictors of outcome. A *P*-value of <.05 was defined as statistical significance.

## 3 Discussion

Knee osteoarthritis is the most common chronic degenerative joint disorder in the clinical, which causes arthritic symptoms, such as joint pain, stiffness, limitations in movement, and loss of functions. Worldwide estimates are that 9.6% of men and 18.0% of women aged over 60 years have symptomatic osteoarthritis. Recently, ESWT was reported to have good results for treating knee osteoarthritis. It provided another alternative for the treatment of knee osteoarthritis. This retrospective study aimed to compare the efficacy of ESWT treatment with sham-ESWT on pain, walking speed, physical function, and adverse effects in knee osteoarthritis. The hypothesis was that ESWT would be an effective treatment for improving pain and physical function in knee osteoarthritis to control symptoms.

## Author contributions

**Conceptualization:** Xianfei Xie.

**Data curation:** Jialing Zhu.

**Formal analysis:** Xianfei Xie, Jialing Zhu.

**Funding acquisition:** Hao Zhang.

**Investigation:** Xianfei Xie, Jialing Zhu.

**Methodology:** Hao Zhang.

**Resources:** Hao Zhang.

**Software:** Jialing Zhu.

**Supervision:** Hao Zhang.

**Validation:** Jialing Zhu.

**Visualization:** Jialing Zhu.

**Writing – original draft:** Xianfei Xie, Jialing Zhu.

**Writing – review & editing:** Hao Zhang.
